# Small Imaging Depth LIDAR and DCNN-Based Localization for Automated Guided Vehicle †

**DOI:** 10.3390/s18010177

**Published:** 2018-01-10

**Authors:** Seigo Ito, Shigeyoshi Hiratsuka, Mitsuhiko Ohta, Hiroyuki Matsubara, Masaru Ogawa

**Affiliations:** Department of System & Electronics Engineering, Toyota Central R&D Labs., Inc., 41-1, Yokomichi, Nagakute, Aichi 480-1192, Japan; hiratuka@mosk.tytlabs.co.jp (S.H.); ohtam@mosk.tytlabs.co.jp (M.O.); hmatsu@mosk.tytlabs.co.jp (H.M.); ogawa@mosk.tytlabs.co.jp (M.O.)

**Keywords:** single-photon avalanche diode, light detection and ranging, imaging LIDAR, deep learning, deep convolutional neural network, localization, sensor fusion

## Abstract

We present our third prototype sensor and a localization method for Automated Guided Vehicles (AGVs), for which small imaging LIght Detection and Ranging (LIDAR) and fusion-based localization are fundamentally important. Our small imaging LIDAR, named the Single-Photon Avalanche Diode (SPAD) LIDAR, uses a time-of-flight method and SPAD arrays. A SPAD is a highly sensitive photodetector capable of detecting at the single-photon level, and the SPAD LIDAR has two SPAD arrays on the same chip for detection of laser light and environmental light. Therefore, the SPAD LIDAR simultaneously outputs range image data and monocular image data with the same coordinate system and does not require external calibration among outputs. As AGVs travel both indoors and outdoors with vibration, this calibration-less structure is particularly useful for AGV applications. We also introduce a fusion-based localization method, named SPAD DCNN, which uses the SPAD LIDAR and employs a Deep Convolutional Neural Network (DCNN). SPAD DCNN can fuse the outputs of the SPAD LIDAR: range image data, monocular image data and peak intensity image data. The SPAD DCNN has two outputs: the regression result of the position of the SPAD LIDAR and the classification result of the existence of a target to be approached. Our third prototype sensor and the localization method are evaluated in an indoor environment by assuming various AGV trajectories. The results show that the sensor and localization method improve the localization accuracy.

## 1. Introduction

**Figure 1:**
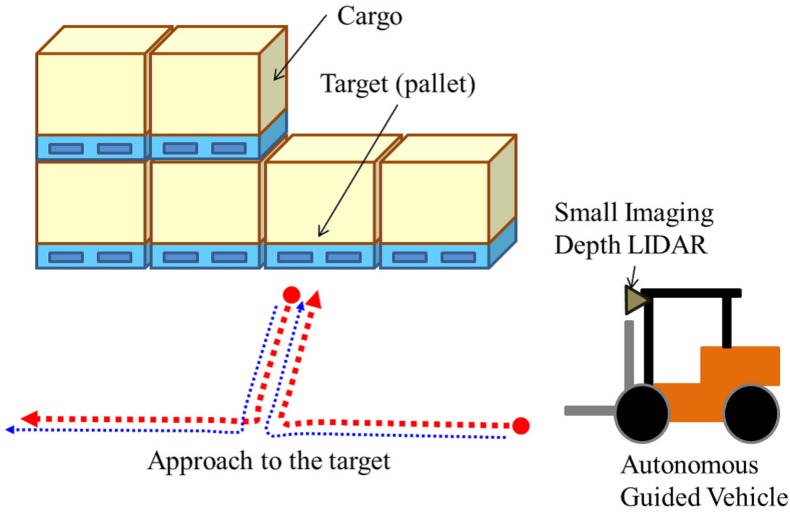
Example of an assumed scenario for Automated Guided Vehicles (AGVs). An AGV equipped with a small imaging depth LIDAR automatically approaches a pallet and transports the pallet to another location.

Automated Guided Vehicles (AGVs) constitute important technology in modern factories, in which installation of automated logistics systems has begun. The main functions of an AGV are its own localization and the detection of a target, for example a pallet or cargo (see Figure 1). In the case of a large factory, we assume that typical features for localization such as walls, pillars and static facilities tend to be positioned distant from each other. For precise localization, a sensor for AGV application must measure these targets over a long range. In addition, in many scenarios, an AGV moves in the daytime and at night, both indoors and outdoors. Therefore, a sensor for AGV application must have the capability of target distance measurement under many lighting conditions. To this end, LIght Detection and Ranging (LIDAR) in combination with an imaging sensor such as a camera yields particularly useful sensors in various scenarios. In addition, the fusion of these sensor outputs is helpful for precise localization.

LIDAR [1,2] is necessary to support the capabilities of AGVs and autonomous cars. An extremely popular LIDAR product for these vehicles is that produced by Velodyne [1]. Velodyne sensors output approximately one million points with $360^{\circ }$ Fields Of View (FOVs) using multiple laser transmitters and receivers. However, the need for multiple transmitters and receivers makes it difficult to incorporate sensors at low cost and to develop small-sized sensors. A fusion-based approach, in which the output from various sensors is fused, is useful for robust and precise localization. Practical approaches to sensor fusion [3,4] have been studied for a long time. In addition to those investigations, we have also previously explored the basic performance of fusion-based localization, as reported in conference proceedings [5]. When a system fuses data from LIDAR with those obtained by capturing images and from other sensors, the system requires an external calibration procedure among the different sensors. Although accurate calibration is necessary for precise localization, this process requires continuous additional effort. Recently, Deep Convolutional Neural Networks (DCNNs) have achieved remarkable performance for many tasks [6,7,8]. However, it remains difficult to estimate the capabilities of the method and to determine an optimal network structure for each task in advance. Zoph and Le [9] and Real et al. [10] have attempted to overcome these difficulties to automatically explore an optimal network architecture, but this problem remains unresolved. Therefore, for now, it is necessary to explore the performance of the DCNN-based method for each type of sensor and task individually.

**Figure 2:**
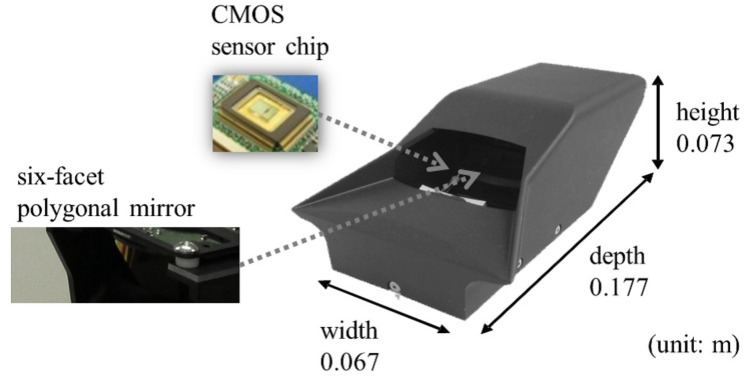
Third prototype of our LIDAR, having only one laser diode and one sensor chip. This feature enables a small-sized prototype. The size is similar to that of a small 500-mL plastic bottle, and the LIDAR has a six-facet polygonal mirror.

**Figure 3:**
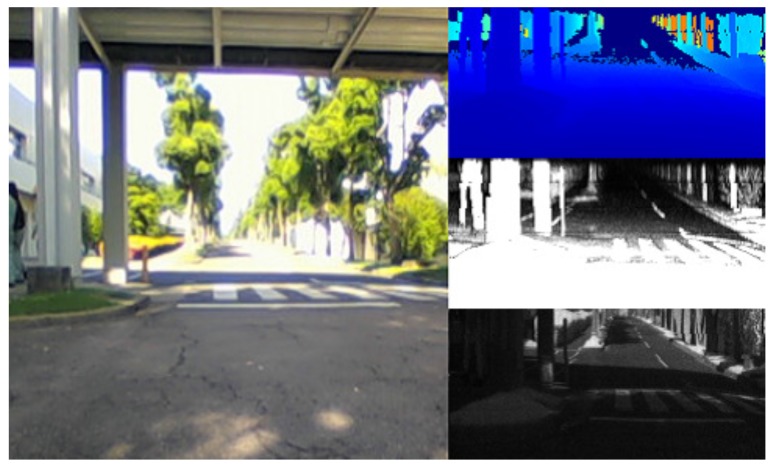
Examples of the output of the prototype LIDAR with the reference image in an outdoor environment: (right, top) range image data; (right, center) peak intensity image data; (right, bottom) monocular image data; (left) reference image captured by the camera.

In this paper, we address these issues based on our third prototype sensor. Our small sensor, named “Single-Photon Avalanche Diode (SPAD) LIDAR,” has only one laser diode and a one-chip detector; this structure enabled us to develop a compact prototype (see Figure 2). In addition, this structure is also expected to inspire a low-cost LIDAR design in the future. The sensor simultaneously outputs range image data, peak intensity image data, and monocular image data (see Figure 3). The sensor outputs these three types of data using the one-chip detector. Therefore, the outputs of the sensor share the same coordinate system, and external calibration among outputs is not required. This calibration-less structure is very helpful in real scenarios in which AGVs are employed. Using the SPAD LIDAR, we developed a DCNN-based localization method, SPAD DCNN, which was designed to fuse the range image data, monocular image data, and peak intensity image data.

The presentation of our results proceeds as explained below. Section 2 introduces related work. Section 3 presents an overview of the SPAD LIDAR. Section 4 explains the localization method using SPAD LIDAR and DCNN. Section 5 presents an evaluation of our localization technique in an indoor environment, with conclusions being presented in Section 6.

## 2. Related Work

In the past decade, considerable progress has been made in the development of 3D cameras [11,12,13,14,15,16,17]. In particular, 3D cameras from Microsoft and Canesta [11,12,13] have had considerable impact on many consumer applications. Micro Photon Devices (MPD) [14] has launched a single-photon counting device, and PMD Technologies [15] has developed a 3D camera with a 35-m range. Among academic institutes, the Swiss Federal Institute of Technology in Lausanne (EPFL) [16] has developed an SPAD-based 3D camera using Complementary Metal-Oxide Semiconductors (CMOS). Work by Fondazione Bruno Kessler/Integrated Radiation and Image Sensors (FBK/IRIS) [17] has developed a 3D camera based on indirect Time-Of-Flight (TOF) imaging using CMOS. In AGV and autonomous vehicle applications, long-range capability is very important for robust localization and safety. For example, in the case of a large factory, typical localization features such as walls or pillars are likely to be positioned at great distances. If the range capability is low, it is difficult to localize the vehicle itself. In addition, an AGV moves both indoors and outdoors under many lighting conditions. Therefore, in this study, we attempted to develop long-range 3D imaging LIDAR with fusion-based localization. 3D LIDAR [1,2] has become popular for use in AGV and autonomous car applications. In the Defense Advanced Research Projects Agency (DARPA) Urban Challenge, many teams [18,19,20] used the Velodyne HDL-64 [1] LIDAR, which has 64 laser transmitters. The Velodyne LIDAR is one of the most commonly-used LIDARs for AGVs and autonomous cars. Although HDL-64 is advantageous in that it outputs a dense point cloud, the sensor size is large. In 2011, Velodyne launched a small LIDAR, named HDL-32, which has 32 laser transmitters. This sensor is approximately the size of a soda can. The latest LIDAR by Velodyne is the VLP-16, with 16 laser transmitters, which is of a similar size to our prototype. However, VLP-16 still has many laser transmitters and receivers. In contrast, the small 3D LIDAR proposed by Kimoto [2] uses a single pair of sensors consisting of a laser transmitter and receiver. That LIDAR mechanically rotates a resonant mirror and reconstructs 8000 points at 20 Hz. Because of this mechanical structure, the point cloud shape is unique.

Marked interest has arisen in sensor fusion for localization and recognition [3,4]. Maddern and Newman [3] have fused Velodyne VLP-16 LIDAR data and stereo data from the Bumblebee XB3 using a probabilistic approach in real time. Further, Mees et al. [4] have fused different sensor modalities using a mixture of convolutional neural networks for object detection in a changing environment. These systems require external calibration among their sensors.

Numerous studies have investigated LIDAR-based, image-based and fusion-based localization. Recently, DCNN revolutionized robotics applications [6,7,8,21,22,23] such as localization, mapping, recognition and semantic segmentation. Hence, Goeddel and Olson [6] classified semantic place labels from an occupancy grid map. That method employs 2D range data as the network input and outputs the place type (e.g., room, corridor or doorway). Further, Costante et al. [7] used DCNN to estimate visual odometry. That method employs a dense optical flow, which is extracted from consecutive images as input and which finally outputs the motion of a camera. In addition, Arroyo et al. [21] used DCNN for topological position localization. Their method fuses the image information from multiple convolutional layers. PoseNet, developed by Kendall et al. [22], estimates a camera pose relative to an arbitrary coordinate frame. PoseNet has two output layers: a regression layer for position and a regression layer for orientation. That method is end-to-end trained to regress the camera position and orientation. Finally, Walch et al. [23] combined CNN and Long Short-Term Memory (LSTM) for camera pose regression in indoor and outdoor environments. Their methods concatenate a fully-connected CNN layer to the LSTM unit and improve the localization error.

## 3. SPAD LIDAR

**Figure 4:**
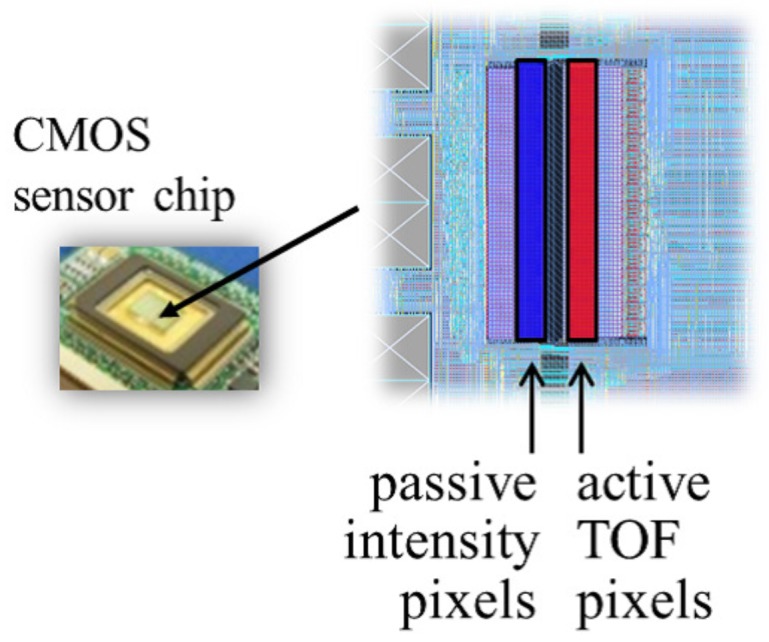
Prototype sensor chip.

**Figure 5:**
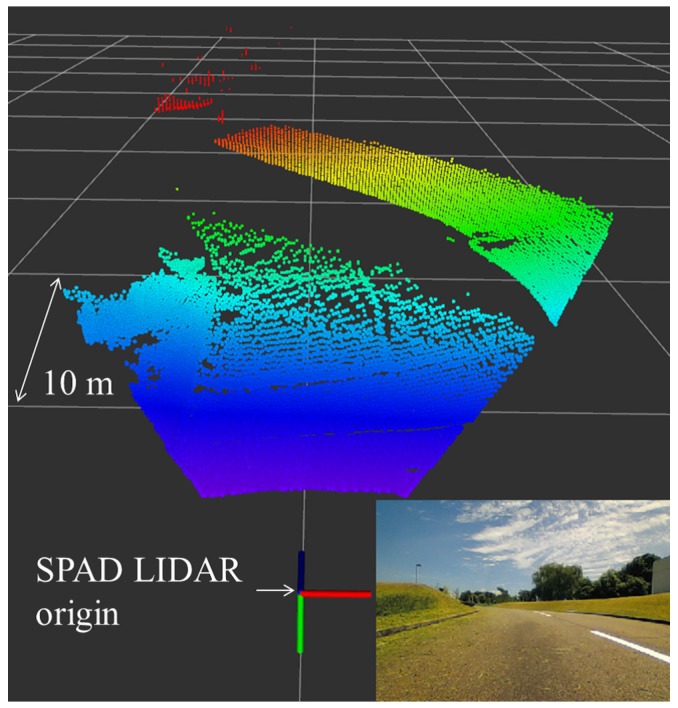
Dense point cloud of Single-Photon Avalanche Diode (SPAD) LIDAR in an outdoor road environment.

The SPAD LIDAR is a laser range sensor based on the TOF method and an SPAD. The SPAD generates accurately-timed digital trigger signals upon detecting light with extremely low optical power [24]. Work by Niclass et al. [16] and Rochas et al. [25] has enabled use of SPAD arrays for imaging. We have also presented our first and second prototypes of the SPAD LIDAR in recent previous papers [26,27]. Based on our SPAD technology, we here report the development of the third prototype of our SPAD LIDAR, as shown in Figure 2. The third prototype primarily improves the overall LIDAR size. In particular, the height of the third prototype is approximately 30% less than those of the previous prototypes. The prototype has a six-facet polygonal mirror that rotates to perform single-beam laser scanning with horizontal and vertical FOVs of 55${}^{\circ }$ and 9${}^{\circ }$, respectively (see Figure 2). The sensor has two SPAD arrays to measure both laser and environmental light on the same sensor chip (see Figure 4). These two arrays are not identical, but the SPAD specifications are the same. The SPAD arrays on the right measure the TOF of the laser beam for active imaging, whereas the SPAD arrays on the left measure environmental light for passive imaging. Both SPAD arrays are rigidly installed on the same sensor chip. Based on this structure, the sensor does not require external calibration between range data and monocular data, which is very helpful for real AGV systems. Although it uses only a single pair comprised of a laser transmitter and receiver, our device can acquire a dense point cloud (see Figure 5). The appearance of dense point clouds in Figure 5 is similar to that of a typical RGB-D sensor such as Kinect [13]; however, our sensor can acquire range data at up to 70.0 m. Furthermore, SPAD LIDAR simultaneously outputs three kinds of data: range image data, peak intensity image data and monocular image data. The range image data indicate the distance between the sensor and an object. The peak intensity image data represent the certainty of the range data. The monocular image data indicate the amount of environmental light. Table 1 presents the main specifications of the SPAD LIDAR. From this table, SPAD LIDAR can perform 70-m measurements, which is a long range for an SPAD detector. Usually, the SPADs are triggered at a shorter distance by background illumination. To prevent this problem, a time-correlation trigger technique is implemented in the SPAD LIDAR [26].

We have also developed a SPAD LIDAR software package for the Robot Operating System (ROS) [28], which is an open-source, meta-operating system for robots. The SPAD package outputs SPAD LIDAR data as ROS topic data, and the SPAD LIDAR parameters are controllable via ROS. We use the SPAD LIDAR output as the input for DCNN-based localization.

## 4. SPAD DCNN

This section introduces our SPAD DCNN localization method based on the SPAD LIDAR and DCNN. Our localization method is based on supervised learning, which is a machine learning task, where functions are inferred from inputs and supervised outputs. An inferred function consists of a set of parameters; here, we refer to these inferred functions as the DCNN model. In accordance with the supervised learning procedure, our DCNN-based localization mainly consists of two steps. One is a training step in which the DCNN model is created, and the other is the estimation step for localization. In the training step, the method creates a DCNN model using the SPAD LIDAR data as input and the supervised position data as output. The supervised position data are acquired by a motion capture system. In the estimation step, the method conducts localization using only the SPAD LIDAR data with the DCNN model.

**Figure 6:**
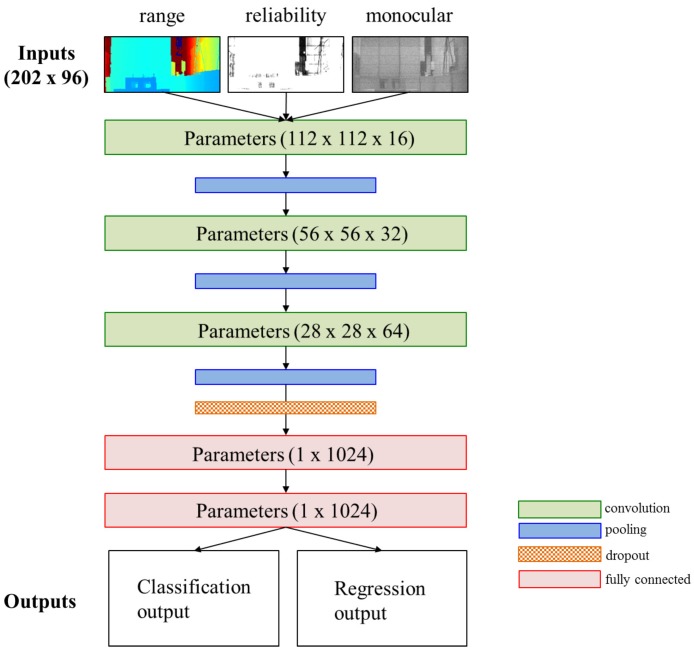
SPAD DCNN model.

Figure 6 shows our DCNN model, which consists of three convolution operations, three pooling operations, one dropout operation, two fully-connected operations and two kinds of outputs. Detailed descriptions of each operation are given in [29]. The DCNN input is our SPAD LIDAR data. The size of each input is 202 × 96 pixels. The DCNN has two kinds of output. One set of output data is the classification results. The classification task of this model is to classify whether a target is in sight of the FOV. For example, in an AGV application, the target is a pallet that the AGV is required to approach. The other output data are the regression results. The regression results are the 3D position and orientation of the SPAD LIDAR.

For this multi-output structure, our DCNN employs the multi-task loss function presented in [30,31]. The loss function for our DCNN is defined as:

(1) $$Loss=L_{cls}\left(p,u\right)+\lambda \left[u\geq 1\right]L_{reg}\left(t^{u},v\right),$$

where $L_{cls}\left(p,u\right)$ represents the classification loss for the true class *u* and *p* is an estimated class. If there is a target in the input data, the true class becomes one. We use cross-entropy for $L_{cls}\left(p,u\right)$. Additionally, $L_{reg}\left(t^{u},v\right)$ is the regression loss for a predicted position and orientation $t^{u}=\left(t_{x},t_{y},t_{z},t_{qx},t_{qy},t_{qz},t_{qw}\right)$ of the SPAD LIDAR. Further, *v* is the ground truth position and orientation. We use the mean squared error for $L_{reg}\left(t^{u},v\right)$, and λ, the value of which is empirically decided, controls the balance between the classification loss and regression loss. Our DCNN approach minimizes this multi-task loss.

## 5. Experiments

**Figure 7:**
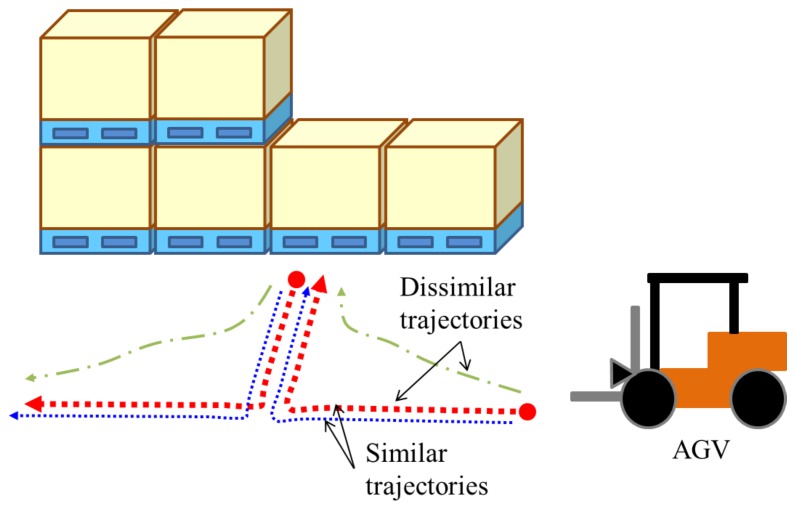
Assumed scenario for evaluation. An AGV moves to the target along various paths. The trajectories are similar in some cases and dissimilar in others.

In this section, we present an evaluation of our sensor and localization method for industrial factory scenarios. Figure 7 shows the assumed scenario of the experiments. In a certain factory, an AGV equipped with the SPAD LIDAR approaches a pallet. The AGV selects and lifts the cargo and then carries the cargo to another location repeatedly. In some cases, the AGV moves along similar trajectories (see the red and blue lines in Figure 7), and in other cases, the AGV follows dissimilar trajectories (see the red and green lines in Figure 7). For practical use, we must explore the basic performance of the DCNN-based localization method with the SPAD LIDAR in both cases.

To this end, we designed the experimental evaluation with similar and dissimilar trajectories to show the following: (a) the ability of the three SPAD LIDAR outputs to boost the localization performance compared to conventional LIDAR, which only outputs range data; (b) the effects of using different trajectories on SPAD DCNN localization. We performed three sets of evaluations. In the first set, we evaluated the benefits of SPAD LIDAR outputs in terms of the localization error for similar trajectories. To this end, we compared the localization error obtained using the SPAD LIDAR outputs to that obtained for localization using the range data that form the output of conventional LIDAR. We used the range data, peak intensity data and monocular data for localization for SPAD DCNN evaluation and only used the range data for the conventional evaluation. The localization error was measured in terms of the mean of the Absolute Trajectory Error (ATE) [32]. The ATE first aligns the two trajectories and then evaluates the absolute pose differences. Equations (2) and (3) show the definition of ATE:

(2) $$F_{i}:=Q_{i}^{-1}SP_{i}$$

(3) $$ATE\left(F_{1:n}\right):=(\frac{1}{n}\sum_{i=1}^{n}||trans\left(F_{i}\right){\left|\right|}^{2}{)}^{1/2}$$

where the $F_{i}$ denote the transition and rotation errors, *P* is the estimated position, *Q* is the ground truth position and *S* represents the transform matrix from *P* to *S*. The function trans() makes it possible to extract only the transitional error from the argument. The second set of experiments evaluated the benefits of SPAD LIDAR outputs in terms of the classification error for similar trajectories. In the third set of experiments, we evaluated both the localization error and classification error for dissimilar trajectories.

**Figure 8:**
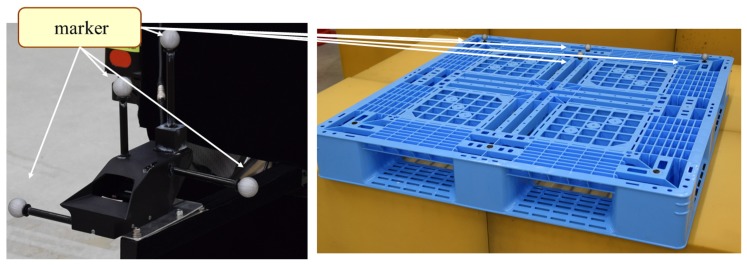
Experimental setup: (**left**) SPAD LIDAR setup on robotic wheelchair; (**right**) pallet position as the target and origin coordinate for localization.

We collected the outputs of the SPAD LIDAR and the ground truth trajectories in an indoor experimental environment. All trajectories and the target position were collected using the VICON motion capture system [33] with markers (see Figure 8). This motion capture system can track a marker in three-dimensional space with an accuracy of millimeter order. All SPAD LIDAR outputs were associated with ground truth trajectories, which assumed the motion and routes of the AGVs. All experiments were conducted over ROS and TensorFlow [34], which is an open-source software library for machine intelligence and which we used to implement SPAD DCNN.

### 5.1. Experiment 1: Localization Accuracy for Similar Trajectories

**Figure 9:**
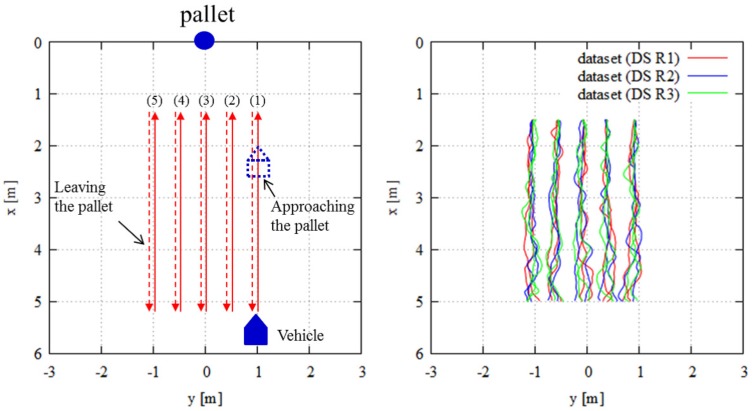
Experimental setup for Experiment 1: (**left**) overview of trajectories; (**right**) ground truth trajectories captured by motion capture system.

This experiment investigated the benefits of SPAD LIDAR outputs compared to those of conventional LIDAR, which employ range data only. Figure 9 (left) shows an overview of the trajectories. These trajectories were assumed to be the typical path of the AGV in the factory. In many cases, the AGV moves along similar paths to perform the task. Therefore, we evaluated the basic localization performance for those trajectories. In this experiment, the vehicle directly approached a pallet and then departed from the pallet repeatedly. In Experiment 1, the pallet was always in sight. Figure 9 (right) shows the ground truth trajectories collected by motion capture. We used Datasets 1 (DS R1), 2 (DS R2) and 3 (DS R3) shown in Figure 9 (right). When evaluating “R1”, we used DS R1 for localization error evaluation and DS R2 and DS R3 for the training step to create the DCNN model. The evaluation and training step data for the evaluation of “R2” were DS R2 and DS R1 and DS R3, respectively. For the evaluation of “R3”, DS R3 was used for evaluation, and DS R1 and DS R2 were used for the training step.

**Figure 10:**
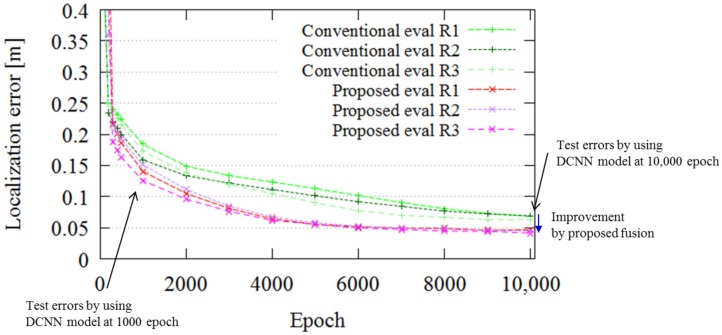
Test error of “SPAD DCNN” localization (red lines) and “conventional” localization (green lines). The SPAD DCNN has a lower test error in all evaluations. The error difference in the 10,000th epoch is improved by fusion of the SPAD LIDAR data.

Figure 10 portrays the localization errors obtained using the two approaches, “Proposed (SPAD DCNN)” and “Conventional”, for all experiments. In Figure 10, one epoch on the x-axis means one step of the training procedure for the entire dataset. The y-axis shows the localization error calculated in terms of the ATE, which is defined in Equations (2) and (3). The ATE evaluates the trajectory consistency, which is important for localizing and controlling the AGV; therefore, we evaluated the localization error in terms of the ATE. The figure presents the effects of SPAD DCNN compared to conventional localization for all evaluations. The curves indicate the progress and convergence of the DCNN model for every 1000 epochs. The final ATE error of the conventional localization at 10,000 epochs is 0.067 m. On the other hand, that for the SPAD DCNN localization is 0.044 m. In other words, use of SPAD LIDAR, which produces three kinds of output, can boost the localization performance. The distance resolution of SPAD LIDAR is 0.035 m (see Table 1); this result is near the resolution limitation. If it is necessary to achieve higher-precision localization in a certain application, we can improve both the SPAD LIDAR hardware and SPAD DCNN software.

### 5.2. Experiment 2: Classification Accuracy for Similar Trajectories

**Figure 11:**
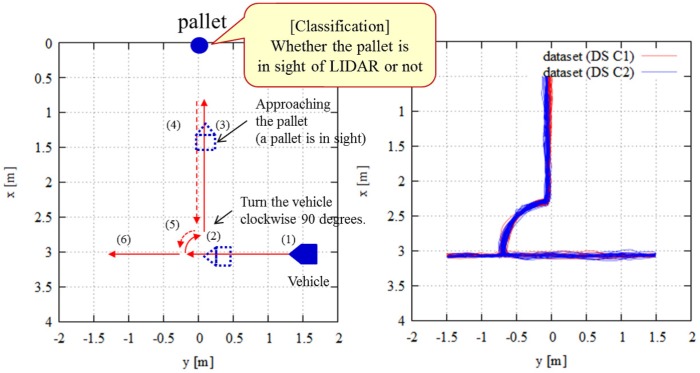
Experimental setup for Experiment 2: (**left**) trajectory overview; (**right**) ground truth trajectories captured by motion capture system.

This experiment evaluated the classification accuracy of the proposed method compared to the conventional method, which only uses range data, in the case of similar trajectories. The proposed method classifies the existence of a pallet. If the pallet is in sight of the LIDAR, the classification result becomes true. Therefore, if the pallet is out of sight, the result becomes false. Figure 11 (left) presents an overview of the trajectories. Initially, the vehicle equipped with the SPAD LIDAR moved in a straight line (see (1) in Figure 11). At this time, the pallet was out of sight of the SPAD LIDAR. Next, the vehicle rotated by 90${}^{\circ }$ in a clockwise direction (see (2) in Figure 11) and approached the pallet. The pallet came within sight during the rotation. The vehicle finally approached the pallet. Figure 11 (right) shows the ground truth trajectories. In this experiment, we collected two datasets: “DS C1” and “DS C2”. Each dataset had 10 similar trajectories containing this route.

**Figure 12:**
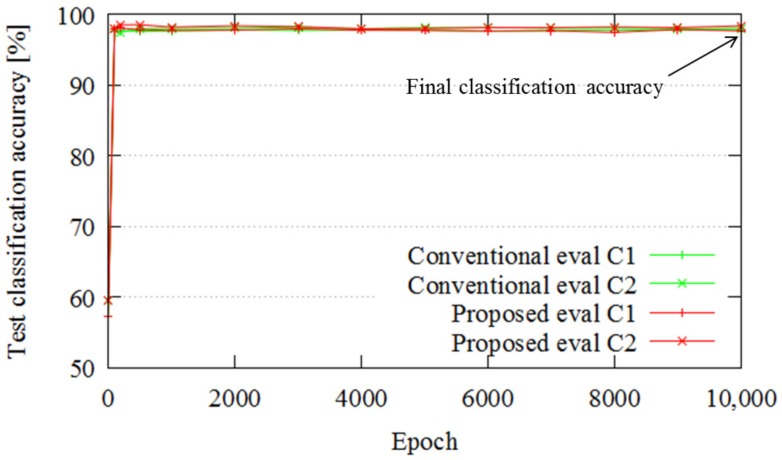
Classification results.

Figure 12 portrays the classification errors for similar trajectories. The classification accuracies of the proposed and conventional methods are 98.04% and 97.96%, respectively. Thus, for similar trajectories, there is no major difference between the proposed and conventional methods in terms of classification accuracy. In the similar trajectory cases considered in Experiment 2, the pallet comes in sight at almost the same point, and the classification task is easy. Therefore, the results with and without fusion are almost the same.

### 5.3. Experiment 3: Localization and Classification Accuracy for Dissimilar Trajectories

**Figure 13:**
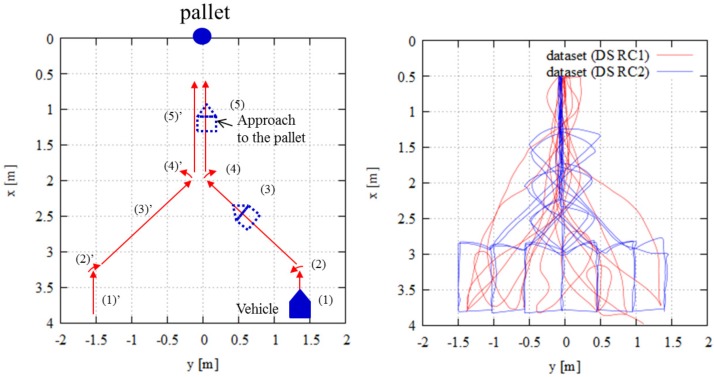
Experimental setup for Experiment 3: (**left**) overview of trajectories of RC2; (**right**) ground truth trajectories captured by motion capture system.

This experiment evaluated the localization and classification accuracy for dissimilar trajectories. We evaluated the basic performance of the DCNN method based on the SPAD LIDAR results obtained in Experiments 1 and 2 in the case of similar trajectories. In a factory scenario, the AGV mostly moves with similar trajectories, but in some cases, the AGV employs dissimilar trajectories. This experiment evaluated the latter scenarios. We collected data for two types of dissimilar trajectories, named “DS RC1” and “DS RC2”. For DS RC1, the vehicle freely approached the pallet (see red line in Figure 13 (right)). For DS RC2, the vehicle moved along a predetermined route (see blue line in Figure 13 (right)). Figure 13 (left) provides an overview of the trajectories in DS RC2. Figure 13 (right) plots the ground truth trajectories in RC1 and RC2.

The evaluation of “RC1” involved use of DS RC1 for test data and DS RC2 for training data, and the opposite for “RC2”.

**Figure 14:**
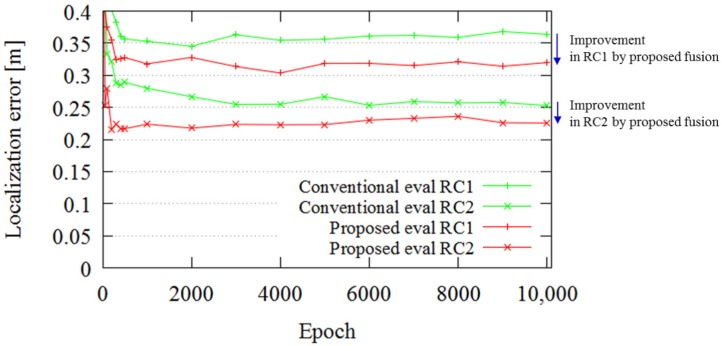
Regression result.

Figure 14 portrays the localization error in the case of dissimilar trajectories. The total average ATE values are 0.31 and 0.22 m for the conventional and proposed approaches, respectively. The localization error is worse than in the case of similar trajectories, for both the proposed and the conventional cases. However, the SPAD LIDAR outputs improve the localization error compared with the conventional method.

**Figure 15:**
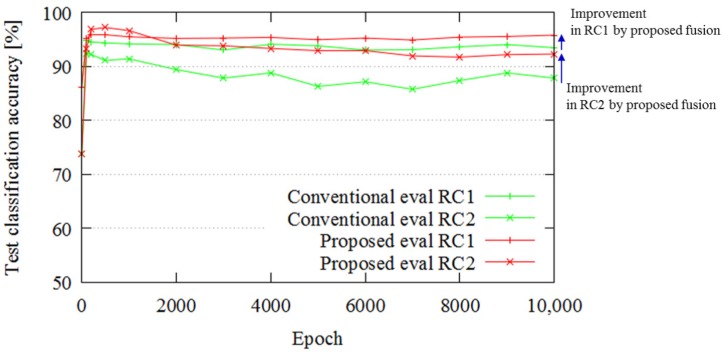
Classification result.

Figure 15 portrays the classification accuracy in the case of dissimilar trajectories. The SPAD LIDAR outputs improve the classification accuracy in this case. Because there are many points at which the pallet comes into sight, the classification task of the Experiment 3 is difficult compared to the case of similar trajectories. Therefore, additional information through fusion helps classification accuracy.

**Figure 16:**
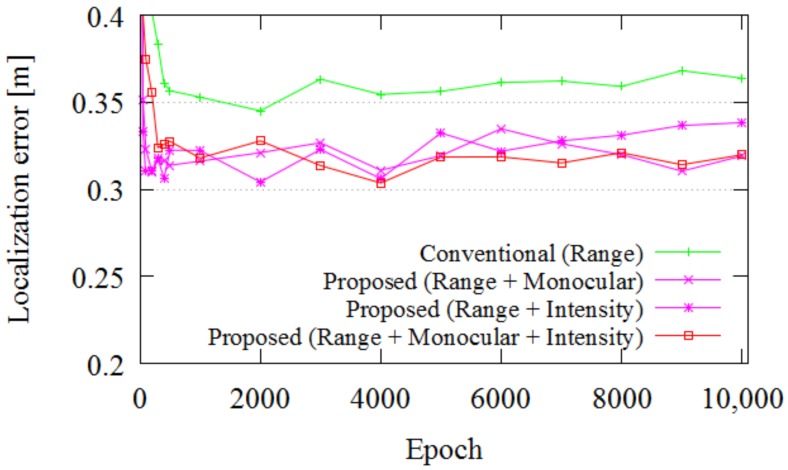
Impact of each SPAD LIDAR data type in Experiment 3.

Finally, we evaluated the impact of each SPAD LIDAR data type for localization. In this experiment, we varied the localization input data. In the first evaluation, only range data were used. In the second and third evaluations, range data with intensity data or monocular data were used. In the fourth evaluation, all SPAD LIDAR outputs were used. In this evaluation, DS RC2 was used as the training dataset, and DS RC1 was used as the test dataset. Figure 16 portrays the impact of each data type on the localization accuracy. As is apparent from the red lines in Figure 16, the three SPAD LIDAR outputs boost the localization performance.

## 6. Conclusions

As described in this paper, we presented SPAD LIDAR and SPAD DCNN, our third prototype sensor and methods for localization, respectively. The main contributions of this paper are the descriptions and analysis of the localization method based on SPAD LIDAR and DCNN. The results show that the output data produced by the SPAD LIDAR improve the localization in terms of accuracy. The small size of our sensor in combination with the novel localization method are useful for autonomous robots, autonomous vehicles and automated guided vehicles. Possible extensions of this work include the exploration of methods for collecting supervised data efficiently. In this study, we used supervised data from motion capture to evaluate the basic performance of SPAD DCNN. In the future, we plan to explore unsupervised or semi-supervised methods with low effort for enhanced practical use.

## Figures and Tables

**Table 1:** Main specifications of the SPAD LIDAR.

	Specifications
Pixel Resolution	202 × 96 [Pixel]
FOV	55 × 9 [deg]
Frame rate	10 [frames/second]
Size	W 0.067 × H 0.073 × D 0.177 [m]
Range	70 [m]
Wavelength	905 [nm]
Frequency	133 [kHz]
Peak power	45 [W]
TOF measurement	Pulse type
Laser	Class 1 laser
Distance resolution	0.035 [m] (short-range mode), 0.070 [m] (long-range mode)
